# Adolescent idiopathic scoliosis screening for school, community, and clinical health promotion practice utilizing the PRECEDE-PROCEED model

**DOI:** 10.1186/1746-1340-13-25

**Published:** 2005-11-30

**Authors:** Timothy A Mirtz, Mark A Thompson, Leon Greene, Lawrence A Wyatt, Cynthia G Akagi

**Affiliations:** 1 Department of Health Sport and Exercise Science, University of Kansas, Lawrence, Kansas; 2 Division of Clinical Sciences, Texas Chiropractic College, Pasadena, Texas

## Abstract

**Background:**

Screening for adolescent idiopathic scoliosis (AIS) is a commonly performed procedure for school children during the high risk years. The PRECEDE-PROCEDE (PP) model is a health promotion planning model that has not been utilized for the clinical diagnosis of AIS. The purpose of this research is to study AIS in the school age population using the PP model and its relevance for community, school, and clinical health promotion.

**Methods:**

MEDLINE was utilized to locate AIS data. Studies were screened for relevance and applicability under the auspices of the PP model. Where data was unavailable, expert opinion was utilized based on consensus.

**Results:**

The social assessment of quality of life is limited with few studies approaching the long-term effects of AIS. Epidemiologically, AIS is the most common form of scoliosis and leading orthopedic problem in children. Behavioral/environmental studies focus on discovering etiologic relationships yet this data is confounded because AIS is not a behavioral. Illness and parenting health behaviors can be appreciated. The educational diagnosis is confounded because AIS is an orthopedic disorder and not behavioral. The administration/policy diagnosis is hindered in that scoliosis screening programs are not considered cost-effective. Policies are determined in some schools because 26 states mandate school scoliosis screening. There exists potential error with the Adam's test. The most widely used measure in the PP model, the Health Belief Model, has not been utilized in any AIS research.

**Conclusion:**

The PP model is a useful tool for a comprehensive study of a particular health concern. This research showed where gaps in AIS research exist suggesting that there may be problems to the implementation of school screening. Until research disparities are filled, implementation of AIS screening by school, community, and clinical health promotion will be compromised. Lack of data and perceived importance by school/community health planners may influence clinical health promotion practices.

## Background

The PRECEDE-PROCEDE (PP) model is a planning model that provides structure for applying theories so the most appropriate intervention strategies can be identified and implemented [1,2]. The PP model has been utilized in public health and holds potential for use by school, community and clinical health promotion practice in preventing a number of disorders [3]. Adolescent idiopathic scoliosis (AIS) is defined as a lateral curvature of the spine greater than 10 degrees accompanied by vertebral motion and is the most common spinal deformity affecting children with the most common form being the idiopathic form [4-6].

The clinical diagnosis of AIS has not been assessed using the PP model. The lack of use of the PP model for program planning and implementation for AIS indicates an area of need. Increased public awareness and screening clinics has resulted in an increased number of children referred for orthopedic opinion yet, over-diagnosis and unnecessary treatment occurs [7-9]. Inconsistencies about AIS may be indicative of the potential value that the PP model has in fully evaluating the diagnosis for the purposes of program planning and implementation [9]. The purpose of this research is to study AIS in the school age population using the PP model and its relevance for community, school, and clinical health promotion [1]. Although community, school, and clinical health promotion are arguably distinct in their mission and approach, their commonalities bring them together under one model for analysis.

## Methods

Green and Kreuter's textbook *Health Promotion Planning: an Educational and Ecological Approach *(Table 1) provides a framework for each of the phases involved in the PP model. The PP model guided the literature and pertinent resource search [1]. A literature search using the National Library of Medicine's MEDLINE aided in locating pertinent studies using keywords scoliosis, prevalence, quality of life, and screening, epidemiology, and policy. An evidence-based approach, i.e. the usage of the best available evidence from the scientific literature, was utilized in determining the value of the literature. Where quantitative literature was lacking usage of qualitative reports from what the authors concluded as credible sources were utilized. In areas where insufficient data existed, expert opinion from the author(s) was utilized as directed by the PP model's framework.

**Table 1:** Phases and Descriptions of the PRECEDE-PROCEED model

**Phase**	**Description**
I. Social assessment	Identify and evaluate the social problems which impact the quality of life of a target population
II. Epidemiological assessment	Defined as program objectives which define the target population (WHO), the desired outcome (WHAT), and HOW MUCH benefit the target population should benefit, and by WHEN that benefit should occur
III. Behavioral/environmental assessment	Focuses on the systematic identification of health practices and other factors which seem to be linked to health problems
IV. Educational/ecological assessment	Selection of the factors which if modified, will be most likely to result in behavior change
V. Administration/policy assessment	Analysis of policies, resources and circumstances prevailing organizational situations that could hinder or facilitate the development of the health program; Assessment of the compatibility of program goals and objectives with those of the organization and its administration and its fit into the mission statements, rules and regulations.
VI. Implementation of the program	
VII. Process evaluation	Used to evaluate the process by which the program is being implemented.
VIII. Impact evaluation	Measures the program effectiveness in terms of intermediate objectives and changes in predisposing, enabling, and reinforcing factors.
IX. Outcome evaluation	Measures change in terms of overall objectives and changes in health and social benefits or the quality of life. It takes a very long time to get results and it may take years before an actual change in the quality of life is seen.

Source: [1] Green & Kreuter, 1999; [14] Brown, 1999

## Results

Use of keywords scoliosis, scoliosis prevalence, and scoliosis screening along with terms PRECEDE-PROCEED and health promotion found no relevant studies using this model in relation to each other. The literature on social assessment of AIS related to the quality of life (QOL) is limited. Very few studies have approached the long-term effects of AIS as it relates to the person's QOL. Epidemiological reports suggest that AIS is the most common form of scoliosis seen in the childhood population and is the leading orthopedic problem seen in school age children. Behavioral and environmental reports related to AIS tend to focus on finding an etiologic relationship yet this data is confounded by the fact that AIS is not a behavioral diagnosis nor is it perceived as having any direct relationship to behavior. The Health Belief Model, the preferred method of the PP model, has not been utilized in any AIS research. The types of health behaviors that can be appreciated are illness behavior and parenting health behavior. These two behaviors may be identified with AIS in the attempt to promote positive self-esteem and good posture. The educational diagnosis is confounded because AIS is considered an orthopedic disorder instead of being related to a specific behavior. The administration and policy diagnosis is compromised by the fact that scoliosis screening programs have been determined to not be cost-effective. There is a wide variance of opinion of what the cost is to screen a child to rule out AIS. Policies are determined in some schools as based on the fact that in the United States, only 26 states mandate school scoliosis screening. The policy diagnosis is confused due to the varying opinions from professional research societies as to when to perform AIS screening. Furthermore, there exists the potential for error with the most common screening tool (Adam's test) resulting in over-diagnosis and inappropriate referral. Overall, the utilization of the PP model demonstrated where gaps exist in the research record.

## Discussion

Throughout school health, community health, and clinical health practice, the quality of health care delivery needs to be provided within a framework using structure, process, and outcome measures [1]. The PRECEDE-PROCEED (PP) model was developed as a planning framework from which health and health promotion programs could be designed [1]. The PRECEDE model is based on the premise that an education diagnosis should precede an intervention just as a medical diagnosis precedes a treatment plan [10]. PRECEDE stands for "predisposing, reinforcing, and enabling factors in educational diagnosis and evaluation." PROCEED stands for "policy, regulatory, and organizational constructs in education and environmental development." This aspect of the model acknowledges the importance of environmental factors in determining behaviors [10]. The PP framework defines intervention development as a systematic process involving nine phases with the first five (social diagnosis, epidemiological diagnosis, behavioral and environmental diagnosis, educational and organizational diagnosis, and administrative diagnosis) involving the identification of health problems and their determinants through a series of diagnostic steps [11]. The PP model can be a useful guide to facilitate the characterization of the resources, educational and behavioral barriers, and organizational factors in a community and can serve to ensure that interventions and other learning opportunities are tailored to the needs and cultural values of the community [12]. The PP model can provide a useful way to organize research varying from clinician attitudes, beliefs, knowledge, and skills to a model for program planning, serving as a framework for the planning and implementation of health education activities aimed at priority areas [11,13]. In summary, the PRECEDE-PROCEED model begins with the outcome of interest and is used to design an intervention for achieving the desired outcome [10].

### Social assessment of AIS

Phase I of the PP model focuses on the identification and evaluation of a possible social problem, which may influence the quality of life (QOL) of the target population [14]. Quality of life is defined as the perception of individuals/groups that their needs are being satisfied and that they are not being denied opportunities to pursue happiness and fulfillment [1]. Bridwell et al reinforced the idea that AIS is of great concern to parents [15]. The parental concern was about surgery creating neurological defect, to reduce future pain, and disability as an adult [15]. Parental concern appeared to be potential dissatisfaction to see their child spend the rest of their life "as is" [15]. Cosmesis has been an important factor to consider in the treatment of adolescent patients with scoliosis [16,17]. Nonetheless, Edgar and Mehta noted that while surgery can improve appearance of patients with AIS, some patients were not always completely satisfied with the cosmetic result [18].

Untreated scoliosis affects the quality of life and can be a disabling disease in the adult [19]. Although back pain is a prominent concern, especially after age 30, the majority of adults are embarrassed by their deformity if left untreated [19]. Patients with AIS ten years after an orthopedic referral have experienced difficulty in lifting, walking and socializing [20]. Women, in particular, are less likely to marry if deformity is not corrected [19]. Females who have been given the diagnosis of AIS during adolescence have a poorer overall perception of health than do women without such a diagnosis [21]. Approximately 9% of girls will discontinue therapeutic brace wearing because of psychological distress related to the deformity around the hips [16]. Ryan and Nachemson discovered that scoliosis is more common in higher socioeconomic groups and indicates AIS as an equal opportunity disorder [22].

It has been suggested that AIS may lead to multiple physical and psychosocial impairments depending on its severity [23]. Previous studies have only assessed generic health measures, functional status, body image, and self-image [23]. Before 2001, no results of long-term outcomes in terms of health-related QOL had been executed for patients treated for AIS [24]. Most research had been directed to determine whether pain becomes a problem with significant scoliosis in individuals as they age [25].

Climent and Sanchez in their study of adolescents with spinal deformities contended that QOL variables include the Risser sign, clinical diagnosis, duration of brace treatment, and degree of correction [26]. These variables do not constitute a significant measurement of patient well-being, are more related to the diagnostic evaluation and do nothing to alter one's perception of happiness. Health educators, school nurses, and clinicians need to be aware of social well-being factors, and how these factors relate to psychosocial functioning.

### Epidemiological assessment of AIS

The epidemiological diagnosis of the PP model constitutes phase II of the PRECEDE portion [14]. The primary task in this phase is to determine for a given target population which health problems, measured objectively, pose the greatest threat to health and QOL [1]. Planners use epidemiologic data to identify and rank the health problems [1]. This ranking is critical for planners because there is rarely enough resources to deal with all or multiple problems [14].

Scoliosis is classified by its etiology [27]. The most common form is the idiopathic form because no cause has been determined or associated with an already existing pathological state. Table 2 lists the classification of scoliosis by etiology. Idiopathic scoliosis can be classified into categories as based on age (Table 3). AIS is present in 2 to 4% of children between 10 and 16 years of age [6]. Classification of AIS can be based on the patient's age at onset, the rate of the curve progression as measured via radiographic by the Cobb angle, the magnitude of the scoliosis at the end of the growth phase, and the curve pattern [6].

**Table 2:** Types of scoliosis

1. Idiopathic scoliosis
2. Neuromuscular scoliosis
3. Congenital scoliosis
4. Neurofibromatosis
5. Connective tissue scoliosis
6. Osteochondrodystrophy
7. Metabolic scoliosis
8. Non-structural scoliosis

Source: [27] Hu et al, 2000

**Table 3:** Types and age range of adolescent idiopathic scoliosis (AIS)

1. Infantile (under age 3 years of age)
2. Juvenile (from 3 to 10 years of age)
3. Adolescent (from 10 years of age to skeletal maturity)
4. Adult

Source: [27] Hu et al, 2000

**Table 4:** Epidemiology of AIS

Prevalence (curve of 10 degrees)	1.7% 1436 of 82,901 subjects
Prevalence (curve of 10–19 degrees or more)	1.5% 1255 of 82,901 subjects
Girls to boys (overall)	2.1:1
Girls to boys (curves less than 10 degrees)	1.5:1
Girls to boys (curves 10 to 19 degrees)	2.7:1
Girls to boys (curves 20 to 29 degrees)	7.5:1
Girls to boys (curves 30 to 39 degrees)	5.5:1
Girls to boys (curves 40+)	1.2:1
Most common curve of at least 10 degrees	Thoracolumbar (34.3%) (n = 493)
Second most common curve	Lumber (33.1%) (n = 475)
Third most common curve	Thoracic (18.2%) (n = 261)
Fourth most common curve	Double curve (14.4%) (n = 207)

Source: [30] Soucacos et al, 2000

**Table 5:** Risk factors for AIS

**Risk factor**	
Curve progression	Female gender Large curve magnitude Skeletal immaturity
Female body type	Much thinner Decreased body weight Decreased chest girth Decreased body mass index

Source: [32] Remes et al, 2001; [39] Sugita, 2000;

Historically, it was believed that the female to male ratio of AIS was 9:1 [28]. Al-Arjani et al found that 59% of all cases of scoliosis were idiopathic with the mean age of discovery at 12.5 years of age [29]. The mean Cobb angle was 58 degrees with 74% of the curves constituting the adolescent type of idiopathic curve with a thoracic curve being the most common with a female to male ratio of 3.8 to 1 [29]. Soucacos et al found that the prevalence of scoliosis was 1.7% of the population with most cases appearing at ages 13 and 14 with a small scoliotic curve in 1.5% of 10 to 19 degrees [30].

The etiology of idiopathic scoliosis may involve genetic, neuromuscular, hormonal, biomechanical, and other abnormalities [31]. The exact pathogenic mechanism of scoliosis is unknown [32]. Three possible etiologic theories still exist; (1) possible bone malformation during development; (2) asymmetric muscle weakness, and (3) abnormal postural control because of possible dysfunction of the vestibular system [33]. These classifications are not mutually exclusive; nor can any of these abnormalities directly produce lateral curves in the spine yet, whatever the underlying etiological factors are they eventually express themselves in the biomechanical changes associated with lateral curve progression [31]. Cummings et al suggested that the etiologic classification system is substantially reproducible but is only moderately reliable [34] while Lenke et al believed that the current system does not appear to have sufficient intra-observer or inter-observer reliability among scoliosis surgeons [35]. However, the diagnosis in pediatrics is directed or established, sometimes exclusively, by an extensive personal and family history and adequate interpretation, which in the end depends on the skill of the clinician [36]. The etiology classification AIS is still in doubt leading to the conclusion that it is multifactorial [37].

The main risk factors for curve progression are: (1) large curve magnitude; (2) skeletal immaturity; and (3) female gender [32]. A high risk of curve progression is usually associated with the following: girls before the onset of menses at around the time of pubertal growth spurt, right thoracic and double curves with magnitude of > or = 30 degrees [30]. Approximately 30% of the scoliotic deformities involve the thoraco-lumbar region whereas 48% and 22% of curves are confined to the thoracic or lumbar spines [38]. Sugita noted that females with scoliosis were much thinner, had a decreased body weight, chest girth, and body mass index [39]. LeBlanc et al supported this finding when it was found that adolescent girls with progressive AIS have a morphologic somatotype (less mesomorphic) that is different from the normal adolescent population [40]. Of adolescents diagnosed with scoliosis, only 10 percent have curves that progress and require medical intervention [6]. Although a small percentage of scoliotic curves undergo progression, the pattern of curve direction and the sex of the child has played a significant role in the ability to differentiate which curves will progress [30].

### Behavioral and environmental assessment of AIS

Phase III of the PP model focuses on the systematic identification of health practices and other related factors which appear to be linked to the identified health problem [14]. Confounding the behavioral and environmental diagnosis of is the fact that the etiology of scoliosis remains unknown in most cases despite extensive research [41]. Two behaviors (Table 6) can readily be identified and include the *illness behavior *and the *parenting health behavior*. Illness behavior is defined as an activity undertaken by an individual, who perceives they are ill, to define the state of their health and discover a suitable remedy [1]. Parenting health behavior is defined as wellness, prevention, at-risk, illness, self-care, or sick-role actions performed by an individual for the purpose of ensuring, maintaining, or improving the health of a child for whom the individual has responsibility [1].

**Table 6:** Health-related behaviors analogous to AIS

**Behavior**	**Definition**
Illness behavior	Activity undertaken by an individual, who perceives they are ill, to define the state of their health and discover a suitable remedy.
Parenting health behavior	Wellness, prevention, at-risk, illness, self-care, or sick-role actions performed by an individual for the purpose of ensuring, maintaining, or improving the health of a child for whom the individual has responsibility.

Source: [1] Green & Kreuter, 1999

These behaviors (parenting health and illness behavior) may be applied to children with AIS. With reference to illness behavior, Frediel et al found that juvenile patients with AIS were unhappier with their lives due to more physical complaints and lower self-esteem [23]. Perceptions of body image, happiness, and satisfaction of adolescents with scoliosis is significant. Danielsson et al noted that those treated for AIS felt they were limited because of difficulties participating physically in activities and/or self-conscious about their appearance [24]. Sapountzi-Krepia et al found that females with scoliosis have a poorer perception of body image [42]. Only 5% of those with scoliosis declared that they had opportunities to discuss their feelings and problems with health professionals, while 90% of the declared that they wanted to have more opportunities to do this [42]. The potential mental anguish a child may endure from AIS is possible because the child can interpret that others perceive him differently as where he/she can see that he/she deviates in posture from his/her peers. The potential problem fully defining this as an illness behavior is the inability of the child to fully define their state of health and thus seek a remedy.

Since AIS is commonly identified in children indicates a parental role (parenting health behavior). Bridwell et al found that when it came to their child about scoliosis surgery the parents concerns were naturally higher and expectations greater than that of the child undergoing the procedure [15]. Table 7, devised by expert opinion since the data is unavailable, establishes the behavioral prioritization and possible treatment focus in AIS. In addition to carefully selected surgical candidates, good family support, a proper educational environment, and promotion of independence at an early age are required to achieve maximal adult function [43]. Interventions should be devised to maintain or improve the person's orthopedic, pulmonary, and functional status as it applies to activities of daily living (ADL). [44] A great deal of experience, patience and the consideration of the patient's individual demands are inevitable for successful treatment [45,46].

**Table 7:** Behavioral prioritization in AIS

**Prevention**	**Treatment**
1. Prevent low self-esteem.	1. Cognitive therapy possibly.
2. Proper compliance if brace treated.	2. Compliance education.
3. Family low self-esteem.	3. Family support intervention.

Growth and proper posture is an important environment in which spinal curves progress and peak prevalence rates occur at the ages of 11 and 13 years [47]. Changes in the intervertebral disc and endplate composition have been implicated as possible etiologic factors in the pathogenesis of AIS suggesting that scoliotic changes are due to an altered and ineffective synthetic response to a pathologic mechanical environment [48]. Proper posture improves QOL by stability in seating and standing by correction of pelvic obliquity and truck instability [49]. However, there is a strong association between back pain and smoking in people with scoliosis suggesting that smoking may have a greater impact on persons with injured spines [50,51]. Although smoking would aptly be described as a behavioral/lifestyle decision, with AIS it can be classified as an environmental factor as per the potential damage to an already injured spine. No studies have assessed adolescent smoking use and/or peer influence on those with AIS. Industrial environmental factors have not significantly influenced the prevalence of AIS [52]. Table 8, since the data is limited and established by expert opinion, offers the environmental prioritization for AIS which offers a possible role of preventive treatment.

**Table 8:** Environmental prioritization in AIS

**Prevention**	**Treatment**
1. Proper posture	1. Spinal education
2. Reduce incidence of delayed menarche	2. Possible role of birth control; athletic education to avoid female triad
3. Proper compliance if brace treated	3. Compliance education of orthosis
4. Infection control if surgically corrected	4. Home education
5. Prevent future back pain	5. Back safety; child back pack education; tobacco prevention/cessation;

### Educational and ecological assessment of AIS

The educational and ecological phase attempts to identify factors that necessitate change to initiate and sustain the process of behavioral change and thus become the immediate targets/objectives of programming intervention priorities [1]. Three areas are important to the educational assessment: predisposing factors, enabling factors, and reinforcing factors [1,14]. Predisposing factors include knowledge, attitudes, beliefs, personal preferences, existing skills, and self-efficacy toward the desired behavior change. Reinforcing factors include factors that reward or reinforce the desired behavior change [6]. Enabling factors are psychological/emotional or physical factors that facilitate motivation to change behavior [6].

The most widely used measure in the PP model, the Health Belief Model, has failed to be utilized in any scoliosis research. The authors cannot account for the lack of research with this model for the diagnosis of AIS. A research question that may need to be asked is "does AIS create behavioral problems? And if so, can the behavior be changed?" The Child Health Questionnaire Outcomes Data Collection instrument and the American Academy of Orthopedic Surgeons Pediatric Outcomes Data Collection instrument may need to be interpreted with a differing view [53]. *Predisposing factors: *AIS may lead to a more negative social affect as based on societal standards from a cosmetic point of view. Since females are mainly effected and society places emphasis on superficial appearance this may preclude towards the negative social affect. *Enabling factors: *Currently, twenty-six states have laws that mandate scoliosis screening, and other states without such laws may still provide state-supported screening programs or have screening programs conducted voluntarily in communities by local agencies [54]. Sugita believed it is necessary for school teachers and school nurses to pay more attention to a student's lifestyles since body mass index, chest girth, and body weight is significantly lower in females with AIS [39]. Yet, those with contact with potential AIS patients need to determine the value the community has for AIS. Those in school health settings need to be aware of mandatory screening statutes. For the patient with AIS and the nurse/clinician it is particularly important to define the early therapeutic prognosis because treatment can be long and difficult [55]. Lantz and Chen found that postural and lifestyle counseling has no discernable effect on the severity of curves as a function of age, initial curve severity, frequency of care, or attending physician [56]. Enabling factors confound the treatment of AIS and/or make difficult any type of positive bio-behavioral change [56]. *Reinforcing factors: *Confounding the reinforcing factor for reward is the negative connotation as being "labeled" as scoliotic thus leading to a psychosocial effect [57]. The need for increasing self-esteem for those afflicted with AIS, as determined from the behavioral assessment, appears validated.

The factors noted previously might help or hinder health behavior(s). The question that needs to be forwarded is "what priorities for intervention(s) should be listed on these issues?" Three areas as it relates to AIS include the components of prevalence, immediacy, and necessity [1]. Prevalence can be answered from the epidemiological assessment, nonetheless, immediacy and necessity needs to be determined. The priorities must take into account what is known as it applies to the categories of importance and changeability.

Table 9, established by expert opinion since the data in this area is unavailable, represents the prevalence, immediacy and necessity of AIS as a clinical diagnosis in the educational assessment. Changeability may be interpreted to not only mean change in the scoliotic presentation by orthosis or surgery but to potential change in negative perception of self. As per necessity, the evidence thus far, points to the mandatory need for screening of AIS in this population. Although learning and resource objectives have not been covered in AIS research, a developmental model may be arbitrarily advanced which targets the three categories of knowledge, beliefs, and skills. Table 10 (established by expert opinion) represents a model covering the target groups and may include not only the person with AIS, but the primary parental care giver, school nurse/health educator, and/or healthcare provider.

**Table 9:** Priorities within categories of AIS

**Importance**	**Priority**
Prevalence	Females, age 9–11; 2 to 4% of general population
Immediacy	When females reach this stage; delayed menarche
Necessity	Mandatory depending on community and parental concern

**Changeability **	**Priority**

Behavior	Parents as caregivers; teachers as health educators; Increased awareness as to potential of problem in age group; social concern via cosmesis;
Scoliotic deviation	Dependent upon severity at time of diagnosis; treatment compliance; cosmetic effect;

**Table 10:** Learning and resource objectives for AIS

Problem: Teaching health educators on AIS;	
Problem: Clinical review for school nurses/physicians;	
Knowledge	Understanding of natural history of AIS Increase appreciation for self-esteem issues as per children Identify high-risk groups
Beliefs	Elimination of prior misconceptions about AIS Development of a proper posture attitude
Skills	Identify and comprehend current policies, statutes, and research concerns Be able to perform the basic screening assessment and be able to utilize such assessments proficiently

### Administrative and policy assessment

Phase V of the PP model takes into consideration the administration and policy aspects. This phase focuses on the administrative and organizational concerns which must be addressed prior to program implementation [14]. Green and Kreuter defined administrative diagnosis as the analysis of polices, resources and circumstances, and prevailing organizational situations that could hinder or facilitate the development of the health program [1].

Bott noted four basic criteria that are important when determining if AIS is amenable to detection and treatment by screening assessment methods. First, the diagnostic test used must be highly sensitive. Second, AIS should cause substantial morbidity or mortality. Third, early detection and treatment should eliminate the condition or prevent its progression. Finally, the incidence of AIS should be high enough to warrant the utilization of time, resources, and money [58].

The most notable policy in AIS pertains to the screening of children while they are in the age-range of vulnerability. Scoliosis detection through screening of school children is a technique that has been popularized over the last three decades and has been instituted in schools during the child's attendance in fifth to ninth grades. This effort was to achieve early detection of progressive AIS [59,60]. However, there is conflict among various authors and professional groups in formulating policy from the research record [61]. Nussinovitch et al concluded that screening programs for school age children coupled with subsequent follow-up procedures are worthwhile [62]. Wong et al suggested that screening of 11- to 12- and 13- to 14-year-old girls can identify a significant number who could benefit from treatment" [63]. The Scoliosis Research Society has recommended annual screening of all children age 10 to 14 years; the American Academy of Orthopedic Surgeons has recommended screening girls at the ages of 11 and 13 years and boys age 13 or 14; the American Academy of Pediatrics has recommended screening routinely at ages 10, 12, 14, and 16 years [64-67]. The Bright Futures guidelines recommend noting the presence of scoliosis during the physical examination of adolescents and children 8 years of age [68]. On the other hand, the US Preventive Services Task Force has concluded that there is insufficient evidence to recommend routine screening [69]. Evidently, there are interpretations from the research record as to what policy recommendations should be enacted. From an administrative perspective, conflicting opinion on recommendations can hinder implementation.

Budgetary concerns for screening of AIS includes the concern for medico-legal issues, cost concerns, outcome measures, and patient preference issues that have not been completely accounted for previously require they be included in a school policy [70]. Health care costs as well as costs in loss of production can increase with the introduction of a clinical screening program [71]. This increase in health care costs can be due to factors such as over-diagnosis, inappropriate referral, and/or misinterpretation of the findings as based on the use of the testing protocol [72,73]. School scoliosis screenings have been reported to cost from as little as 6 cents per child to as much as $194 per child [74-76]. The lower estimate of cost only considered the cost to the school to implement and the higher cost attributed to children with curves of 5 degrees or more [74]. Koukourakis found that the cost of screening each child was estimated to be approximately $10 [38]. Soucacos et al confounded the financial cost figures by suggesting that screening has a negligible cost estimated at 30 cents per child [30]. Renshaw suggested that much valuable data can be secured from screening but costs of screening are not inconsequential and costs in follow-up procedures are high [73]. Table 11 and 12 provide the difference in costs for screening as well as other significant administrative factors. Clearly there is a considerable cost discrepancy and an accurate price tag on school-based screening programs in order that health care systems can allocate resources on a rational basis [77].

**Table 11:** Cost estimate for AIS

**Procedure**	**Cost**
1. Screening per child	US $24.66
2. Child with curve of 20 degrees or more	US $3,386.25
3. Child treated for scoliosis	US $10,836.00

Source: [38] Koukourakis et al, 1997; [74] Yawn & Yawn, 2000; [75] Morais et al, 1985; [76] Lonstein et al, 1982;

**Table 12:** Overview of AIS as it pertains to the administrative diagnosis

**Resource**	**Procedure**
Time	For children starting in the fifth grade
Budget	US6 cents to US$24.66 per child
Personnel	School nurse; physician

Source: [38] Koukourakis et al, 1997

The screening tool of choice has been the Adam's forward bending test which tests for asymmetry via visual inspection [78,79]. Yawn and Yawn noted that school screenings identified some children who went on to receive treatment but referred many more who did not [74]. Challenges in scoliosis screening include the low prevalence rate of clinically significant scoliosis, the inverse relationship of sensitivity and specificity in the screening process because of the poor correlation of clinical deformity and radiographic abnormality, and the inflated cost of these programs because of overreferral [80]. Thus, Type I and Type II errors from the assessment measure can be implicated as the culprit in AIS screening.

Before implementation of a health plan, it is important to know how it fits with the existing organizational mission, policies and regulations and possible recommendations [1]. Brown defined policy diagnosis as the assessment of the compatibility of a program's goals and objectives with those of the organization and its administration thus asking "does it fit into the mission statements, rules and regulations?" [14].

As noted, scoliosis screening is still under debate [81]. In the United States, only 26 states have laws that mandate scoliosis screening, and other states without laws may still provide state-supported screening programs or have screening programs conducted voluntarily in communities by local agencies [54]. In states where there are no mandates, individual schools may formulate policy voluntarily. Individual communities have the ability to decide what is appropriate and feasible for their schools based on the best available data [74].

It has been argued that that scoliosis screening is not cost-effective, but some still favor it as an integral part of preventative medicine [54]. Spinal screening appears to be effective in reducing the need for surgical treatment, but does not decrease the total cost of care for AIS [81]. Administratively, a redefinition of what actually constitutes a "significant" scoliosis for screening as well as the use of objective referral criteria and re-screening patients rather than referring those who have borderline cases can pose problems for local policy development [82]. Nonetheless, an informed physician/school nurse can make this assessment efficiently with a minimum cost to the family and hopefully reduce radiation exposure and an unwarranted referral [83]. The physician should be well aware of the local school's policy as well as the concerns that such a school has as per resources and budget as well as the community's concern. Table 13, established by expert opinion, demonstrates how the PP model can be utilized to ask questions concerning significant inter- and intra-professional factors.

**Table 13:** Policy factors as it pertains to AIS

**Factor**	**Problem**
Intra-professional	Whose recommendation carries more weight? Who is most qualified to perform screenings? Who has the best available data?
Inter-professional	Unknown as research has not compiled political forces. Dependant upon community value/agenda; state law.

Health promotion is inherently political [84]. Notwithstanding, political concerns must be based on accuracy of the research record and to not on unwarranted extrapolations of the research. Higginson demonstrated the politics of AIS screening from a physician's standpoint: "The legislative process is not necessarily a logical one in which good ideas turn into law; rather, success is usually based on relationships, timing, hard work, and luck. Parents also may perceive that screening is effective and insist that their children not be denied something they believe is valuable. Proactive work to educate and change opinion, such as a parent information campaign using the media, PTA's, and school officials can go a long way to reduce or remove potential grassroots opposition" [54].

## Conclusion

Health educator's, school nurses, as well a clinicians awareness of AIS and associated complications may possibly permit more effective patient surveillance, which may afford those at high risk the opportunity for an improved QOL [85]. However, this research using the PP model has found not only gaps in the research but conflicting views as to the value of AIS screening, the time to screen, cost concerns, as well as reliability of the most common screening tool (Adam's test). Despite adequate epidemiological research that suggests a problem the etiology of AIS remains in debate. Although AIS is the most common orthopedic disorder affecting children the previous problems noted also include state mandates and conflicting recommendations from the research record. The physician interested in such service to a school should have knowledge that screenings are ineffective due to examiner error, an assessment tool that is prone to error, and concern that cost-effectiveness for gaining an accurate outcome, as well as professional organizations lack of consistent recommendations exist to aid the decision-making. For effective implementation of a program a detailed evaluation of the pertaining literature and use of expert opinion is needed to fully appreciate the value of the PP model. Further research using the PP model's preference for the Health Belief Model with those afflicted with AIS along with the other gaps found by the PP model may need to become priorities in successfully developing pertinent learning and resource objectives for successful implementation of AIS programs.

## Competing interests

The author(s) declare that they have no competing interests.

## Authors' contributions

All authors contributed equally to this research.
